# A Bayesian Structural Equation Model to Predict Quality of Life in European Older Adults

**DOI:** 10.3390/ejihpe15070127

**Published:** 2025-07-09

**Authors:** José M. Tomás, Aitana Sanz, Zaira Torres

**Affiliations:** Department of Methodology for the Behavioral Sciences, University of Valencia, 46010 Valencia, Spain; aisanzde@alumni.uv.es (A.S.); zaira.torres@uv.es (Z.T.)

**Keywords:** aging, frailty, loneliness, pain, quality of life, Bayesian structural equation model

## Abstract

The ultimate goal of developed societies is to age with quality of life, but this concept is broad, and few studies have addressed which variables specifically affect its dimensions. This study aims to test a model for predicting the impact of frailty, pain, and loneliness on the four dimensions of quality of life (control, autonomy, pleasure, and self-realization). Data were drawn from a sample of 61,355 Europeans from the Wave 7 of the SHARE project, aged 60 or older (M = 71.87, SD = 8.22), from which 55.9% were females. Statistical analyses included a fully Bayesian structural equation model that evidences the significant effect of loneliness, frailty, and pain on the four dimensions of quality of life. The variables have an unequal impact on the different dimensions’ loneliness was the main predictor for the dimensions control, autonomy, and pleasure, while frailty was the main predictor to self-realization. This study supports the need to address these variables to intervene on the different aspects of quality of life in old age.

## 1. Introduction

The World Health Organization (WHO, 2024) defines older adults as individuals aged 60 and above. This demographic group is expected to double by 2050, reaching more than 1.5 billion older adults (UN-Population, 2020). Population aging is particularly pronounced in Europe, where 21.2% of the population was 65 years or older in 2022 (Eurostat, 2023). Europeans are living longer, but with multiple health conditions or mobility limitations in their later years (Eurostat, 2020). As a result, promoting successful aging, characterized by a high quality of life (QoL) has become a central priority in Europe. To achieve this goal, we first need to understand the key factors that influence the quality of life in old age.

In this regard, the Preventive Corrective Proactive (PCP) model, developed by Kahana and Kahana, stands out for its comprehensive approach to understanding successful aging (Kahana et al., 2012; Kahana et al., 2014). The PCP model understands QoL and well-being as the last outcome of the following five components: the self-evaluation of success, life satisfaction, meaning in life, positive affect, and life-value activity (Kahana et al., 2014). The PCP model also considers preventive and corrective strategies, as well as stressors, that influence the QoL of older adults. Preventive actions include health promotion, future planning, and other proactive measures (Petretto et al., 2016). In terms of stressors, the PCP model emphasizes the impact of some geriatric symptoms, such as pain, physical frailty, or sarcopenia among others, have on QoL (Chen et al., 2021; Farrugia et al., 2021; Iglesias-López et al., 2021; Kahana & Kahana, 2003; Lee et al., 2021; Wang et al., 2021).

Geriatric symptoms are conceptualized as a non-pathological clinical condition in older adults characterized by multiple causes that lead to a unified set of manifestations (Cesari et al., 2017). These conditions arise from a complex combination of clinical, psychological, social, and environmental vulnerabilities that are extremely heterogeneous (Inouye et al., 2007). This study will focus on three common geriatric symptoms in later life—frailty, pain, and loneliness—to address the biological, psychological, and social dimensions of aging.

Frailty is considered one of the most significant geriatric symptoms, along with sarcopenia and cognitive impairment (Giné-Garriga et al., 2020). It is the most prevalent, and its main characteristic is the limited ability to cope with stressors due to a decrease in the body’s homeostatic reserve, which increases the risk of falls, hospitalizations, disability, and mortality (Belenguer-Varea et al., 2023; De Luca et al., 2023; Wu et al., 2018). The likelihood of developing frailty is influenced by a range of factors, including natural physiological changes of aging, multimorbidity, malnutrition, the person’s living environment, genetics, social relationships, and personal lifestyle (Belenguer-Varea et al., 2023; De Luca et al., 2023; Jenkins et al., 2023). Frailty has been negatively associated with well-being (Vestjens et al., 2018), life satisfaction (Zapata-Lamana et al., 2022), loneliness (Li et al., 2016; Hoogendijk et al., 2016), limited regular physical and social activity (De Luca et al., 2023), and worse or limited QoL (Belenguer-Varea et al., 2023; Chen et al., 2021).

Pain is a prevalent issue among older adults and affects a significant proportion of this population (Marttinen et al., 2019). In fact, many studies have examined the associations between frailty and pain (Chiou et al., 2018; Guerriero & Reid, 2020; Otones et al., 2019). Systematic reviews and meta-analyses by Saraiva et al. (2018) and Lin et al. (2020) have identified persistent pain as a significant risk factor for the development of frailty. In addition, pain has been shown to predict lower QoL and higher rates of depression (Kaye et al., 2010). In recent studies, such as Bonanni et al. (2023), pain has been associated with worsening QoL due to its strong association with depression. Pain also has been associated with loneliness (Carrasco et al., 2022; Chan et al., 2014; Smith et al., 2019).

Finally, loneliness is recognized as one of the major public health challenges of the 21st century. It is commonly defined as an unpleasant experience due to the contrast between expectations and social relationships (Bandari et al., 2019; Carrasco et al., 2022). Although loneliness can affect individuals across all age groups, it is particularly prevalent among older adults (Conacher, 2017). Several studies have related loneliness to QoL and found a negative impact of loneliness on it (Carrasco et al., 2022; Newman-Norlund et al., 2022).

Although there is a substantial body of literature that has explored the relationships among QoL, frailty, pain, and loneliness, most of the literature tends to conceptualize QoL as a single global indicator or focus on the health-related QoL concept. However, QoL is widely recognized as a complex and multidimensional construct. For that reason, our study adopts a more comprehensive approach by examining QoL through multiple dimensions. This perspective is grounded in the definition proposed by Hyde et al. (2003) and their needs satisfaction model, which incorporates both psychological and subjective well-being, encompassing hedonic and eudaimonic aspects. According to this model, four basic needs (control, autonomy, self-realization, and pleasure) must be satisfied to achieve high QoL in older age.

Control is conceptualized as the capacity to proactively influence the environment, and autonomy refers to an individual’s ability to avoid external interference in their actions (Frias-Goytia et al., 2024; Patrick et al., 1993). Finally, self-realization and pleasure encompass all the dynamic and contemplative processes of being human, with the goal of experiencing a sense of purpose, fulfillment, and happiness (Frias-Goytia et al., 2024; Giddens, 1990; Turner, 1995). The aim of this study is to test a predictive model of QoL in a large sample of Europeans. Specifically, it examines the impact of three latent variables (frailty, pain, and loneliness) on the four dimensions of QoL (control, autonomy, pleasure, and self-realization).

## 2. Materials and Methods

### 2.1. Data and Participants

This study was based on data from the 7th wave of the Survey of Aging and Retirement (SHARE-ERIC, 2024; Börsch-Supan et al., 2013). SHARE is a longitudinal study focused on the study of populations aged 50 and older from Israel and 28 European countries. The SHARE project collects data using computer-assisted personal interviewing (CAPI) and follows a probability-based sampling strategy to obtain representative samples of each participating country; further details can be found in Bergmann et al. (2019). SHARE is guided by international ethical standards, such as the Respect Code of Practice for Socio-Economic Research, and was approved by the ethics council of the Max Planck Society in Munich (Approval Code: 2021_24). The collection of SHARE data and their use are subject to the European General Data Protection Regulation. SHARE data can only be used for scientific purposes and are protected by the German Federal Statistics Act and the German Federal Data Protection Law.

Regarding the sample for this study, we included 61355 Europeans over 60 years old (M = 71.87, SD = 8.22), from which 55.9% were females, and 44.1% were males. Regarding some health indicators, this sample had a mean of 2.08 chronic diseases per person (Median = 2, SD = 1.67), and their self-perceived health, in a 1 (poor) to 5 (excellent) scale, had a mean of 2.63 (median = 3, SD = 1.03). Their economic level, measured as the ability of the household to make economic ends met, was 2.73 (Median = 3, SD = 1.01) recorded in an ordinal scale from 1 (with great difficulty) to 4 (easily).

### 2.2. Measures

To measure the concept and its dimensions, the CASP-12 was employed. This scale has been validated in several studies and used in international research, being the most appropriate and recommended for measuring the degree of coverage of needs and QoL of the older adults (Borrat-Besson et al., 2015; Fekete et al., 2022; Oliver et al., 2021; Pérez-Rojo et al., 2018). The CASP-12 has demonstrated robust psychometric characteristics, establishing it as a suitable multidimensional scale with four correlated factors (Hamren et al., 2015; Pérez-Rojo et al., 2018; Rodríguez-Blázquez et al., 2020). The CASP-12 encompasses the following four dimensions of QoL: control, autonomy, self-realization, and pleasure (Hyde et al., 2003). Answers are given in a Likert scale with four points, from ‘never’ to ‘often’. The internal consistency estimates for each dimension of the scale are ω = 0.78 for control, ω = 0.39 for autonomy, ω = 0.86 for self-realization, and ω = 0.81 for pleasure. The autonomy dimension showed the weakest reliability in our study. This problem has been previously documented in relation to the CASP-12 scale (Borrat-Besson et al., 2015; Oliver et al., 2021; Rodríguez-Blázquez et al., 2020; Sim et al., 2011), and some authors have suggested item modifications or proposed bifactor or trifactor structures (Kerry, 2018; Pérez-Rojo et al., 2018). Despite these concerns about reliability, autonomy remains a key component of QoL in older age. Moreover, the four-dimensional structure of the CASP-12 scale remains widely used. So, in the present study, we opted to retain the four original dimensions of the CASP-12, to facilitate comparability across studies.

Frailty was evaluated using the established measures in SHARE (Romero-Ortuño, 2011; Santos-Eggimann et al., 2009). The assessment included the following: (a) A Mobility Index, derived from the sum of four questions regarding difficulty in activities such as walking 100 m, moving across a room, climbing stairs, and ascending a flight of stairs. Each affirmative response scored 1 point, resulting in a range of 0 to 4, with higher score indicated greater mobility limitations. (b) Appetite, assessed through a binary indicator. Individuals were asked about changes in their appetite, with a score of 1 indicating a “decreased desire for food” and a score of 0 indicating “no decrease in desire for food”. (c) Fatigue, measured with a dichotomous question asking if, in the last month, individuals felt they lacked sufficient energy to engage in desired activities. (d) Vigorous physical activities, assessed by querying the frequency of engaging in vigorous physical activities, with response options ranging from 1 (more than once a week) to 4 (almost never or never). (e) Grip strength, measured twice in each hand using a dynamometer, with the higher of the two values recorded.

Loneliness was measured using the Three-Item Loneliness Scale (Hughes et al., 2004), a short version of the R-UCLA Loneliness Scale by Russell et al. (1980). The scale evaluated the frequency of feelings related to lack of companionship, exclusion, and isolation, with responses on a scale from 1 (hardly ever or never) to 3 (often). The scale showed adequate internal consistency ω = 0.87.

Pain was assessed using two indicators from SHARE. A yes/no question about whether the individual took at least five different drugs per day, and the sum of two yes/no questions related to taking drugs for joint pain or other pain, resulting in a scale ranging from 0 (no pain medication) to 2 (medication for joint or other pain). In this sample, it showed an internal consistency ω = 0.55.

### 2.3. Statistical Analysis

For the purposes of this study, a Bayesian Structural Equation Model (BSEM) was estimated using MPlus 8.11 (Muthén & Muthén, 2017). This model enables the analysis of latent variables or factors (where several indicators are used to measure the construct of interest), such as the dimensions of QoL, frailty, pain, and loneliness. In Bayesian estimation, parameters are treated as random, and the information about them is updated using observed data. Bayesian methods do not rely on asymptotic theory, making them suitable for non-normal data or complex models where traditional parametric methods might not be appropriate (Choi & Levy, 2017; Depaoli, 2021). In this statistical logic, based on the Bayes theorem, the data information (the likelihood function) is combined with prior distributions of the parameters to be estimated to form a posterior distribution for each parameter estimate. This procedure offers good estimates for very complex models or to address possible convergence problems (Kaplan & Depaoli, 2012; Lee & Song, 2004; Muthén & Asparouhov, 2012; Song & Lee, 2012). In Bayesian analysis, ensuring the Markov chain Monte Carlo (MCMC) algorithm converges to the target posterior distribution is crucial. In order to do so, trace plot for each parameter were examined (and showed no convergence problem), as well as R-hat calculated (largest value being 1.018). Therefore, convergence was achieved.

Standardized estimates in the Bayesian model offer the expected change in the outcome in standard deviations when the predictor changes in 1 standard deviation. These standardized estimates allow relative comparisons between estimates and can be interpreted as β < 0.2 = weak effect, 0.2 < β < 0.5 = moderate, and β > 0.5 = strong effect (Acock, 2014).

## 3. Results

Table 1 shows the range, mean, median, and standard deviations of all observed variables involved in the model. The correlations between the observed variables included in the model are presented in the Supplementary Materials (Table S1).

**Figure 1:**
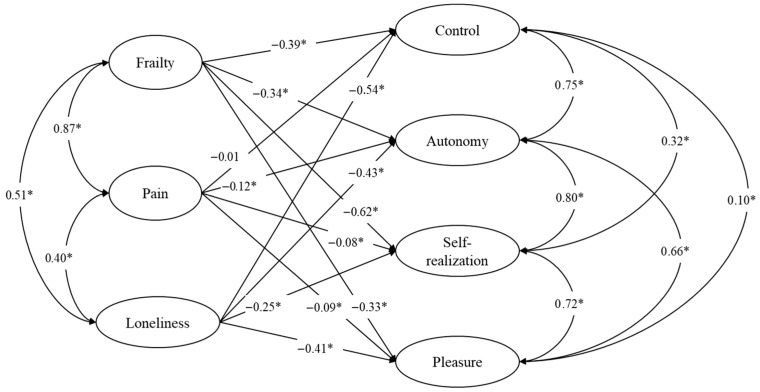
Standardized parameter estimates of the structural model to predict quality of life. Notes: * indicates *p* < 0.05; indicators and errors not shown for clarity.

The full structural model is presented in Figure 1. The model showed good data fit: RMSEA = 0.040, 90% CI [0.040–0.041]; CFI = 0.931. From a statistical point of view, the variables frailty, pain, loneliness, and the four dimensions of QoL are treated as modeled as factors (latent variables). From the theoretical point of view, the model posits frailty, pain, and loneliness as background variables and potential predictors of the QoL’s dimensions.

Regarding the standardized parameter estimates. Frailty, pain, and loneliness were able to predict 64.4% of the variability of control, 59.6% of the variability of autonomy, 55.9% of the variability of self-realization, and 41.6% of the variability of pleasure. Loneliness was the main predictor for the dimensions control (β = −0.54, *p* < 0.01), autonomy (β = −0.43, *p* < 0.01), and pleasure (β = −0.41, *p* < 0.01) and exhibited a significant moderated effect in self-realization (β = −0.25, *p* < 0.01). In this turn, frailty was the main predictor to the dimension self-realization (β = −0.62, *p* < 0.01) and has significant moderate effects in control (β = −0.39, *p* < 0.01), autonomy (β = −0.34, *p* < 0.01), and pleasure (β = −0.33, *p* < 0.01). Finally, pain has a small significant effect on autonomy (β = −0.12, *p* < 0.01), self-realization (β = −0.08, *p* < 0.01), and pleasure (β = −0.09, *p* < 0.01).

All the independent variables included in the model (frailty, pain, loneliness) have significant correlations with each other, particularly demonstrating a strong positive correlation between pain and frailty (r = 0.87, *p* < 0.01). The QoL’s dimensions were also correlated. The correlations that were significant and can be considered large were control with autonomy (r = 0.75, *p* < 0.01); autonomy with self-realization (r = 0.79, *p* < 0.01); self-realization with pleasure (r = 0.72, *p* < 0.01); and autonomy with pleasure (r = 0.66, *p* < 0.01). Table 2 expands on the information included in the figure and shows the prediction effects, the correlation values of the variables, and their corresponding credible intervals, posterior standard deviations, and *p*-values.

## 4. Discussion

The present study aimed to determine the influence of frailty, pain, and loneliness on the dimensions of QoL, which are control, autonomy, pleasure, and self-realization.

Frailty, pain, and loneliness have been extensively studied in scientific literature, indicating a reciprocal relationship between them. Frailty was positively related to loneliness (Li et al., 2016; Hoogendijk et al., 2016) and pain (Guerriero & Reid, 2020; Lin et al., 2020; Otones et al., 2019; Saraiva et al., 2018), negatively with well-being (Vestjens et al., 2018), and with worse or limited QoL (Belenguer-Varea et al., 2023; Chen et al., 2021), as well as physical and social activity (De Luca et al., 2023). Similarly, there are studies associating pain with loneliness, although the results are less conclusive (Carrasco et al., 2022; Chan et al., 2014; Smith et al., 2019), and there are studies indicating a worse QoL (Bonanni et al., 2023; Kaye et al., 2010). Also, there is evidence of a negative impact of loneliness on QoL (Carrasco et al., 2022; Newman-Norlund et al., 2022), as well as on social connections (Bandari et al., 2019; Carrasco et al., 2022).

In our multivariate model, positive and generally large relationships between the four QoL’s dimensions were observed, as expected in the existing literature (Pérez-Rojo et al., 2018; Towers et al., 2015). However, in our model, associations are estimated, while several predictors explain part of their variances, reinforcing QoL as a global and multidimensional construct. Furthermore, the main results obtained showed that frailty, loneliness, and pain were predictors of the different dimensions. These main variables have an unequal impact on the different dimensions of QoL; specifically, loneliness is the most important predictor of control, autonomy, and pleasure, followed by frailty, whereas in the dimension of self-realization, it was frailty that showed the strongest predictor effect. The results could be influenced by the different indicators, since the self-realization dimension asks directly about recent events, while the other dimensions assess situations that deal with both the present and the past. In other words, when faced with a state of fragility that could worsen and even become chronic, future self-realization is more affected.

Pain had a smaller effect on all dimensions, which means that having pain is considered less important when compared to physical (frailty) and social (loneliness) components. However, it is important to acknowledge that these results may be influenced by the way that pain was operationalized in our study. We assessed pain based on participants’ use of pain medication; however, this is an indirect measure that may not accurately reflect the subjective experience or intensity of pain. In contrast, self-report methods are considered the most reliable means of assessing both pain presence and severity (Salaffi et al., 2015). Several validated scales are available for this aim, such as the visual analogue scale (VAS), the verbal rating scale (VRS), and the numerical rating scale (NRS) (Wood et al., 2010; Karcioglu et al., 2018). Future research could use subjective measures to determine if pain has a greater impact on QoL when it is measured with different indicators.

Our study has several strengths, such as the use of a large SHARE project sample, which uses a probabilistic sampling strategy to pick its target population. Moreover, the fact that we used a compilation of representative data from different European countries, specifically from 28 countries and Israel. However, it also has several limitations. Among the general limitations of the study, we can highlight its cross-sectional nature and the impossibility of obtaining information on causality and the direction of the variables over time. Likewise, the self-reported origin of the data contributes to the appearance of various biases, including confirmatory bias and social desirability. In addition, the results may be affected by variance-related aspects of common methods. Despite the procedures carried out to guarantee the reliability and validity of the data from SHARE, individual interviewer differences may generate a series of biases related to the data, which would affect their interpretation. Finally, future research could expand on these findings by incorporating additional variables related to loneliness, pain, frailty, and QoL, such as age, gender, educational level, marital status, and economic situation (Oliver et al., 2021; Rodríguez-Blázquez et al., 2020).

Although many factors can affect QoL in later life, this study focuses on frailty, pain, and loneliness to address the bio-psycho-social dimensions. This choice was made to address the bio-psycho-social aspects of main geriatric syndromes. It is well known that geriatric giants are prevalent and go along with the age increase in older people (Prell et al., 2023), with those selected syndromes having the greatest impact on their functionality and autonomy (Giné-Garriga et al., 2020; Prell et al., 2023). They also have increased morbidity and mortality (Giné-Garriga et al., 2020) and a direct impact on the person’s QoL (Belenguer-Varea et al., 2023; Chen et al., 2021). It is known that loneliness is a risk factor for geriatric syndromes, such as cognitive impairment (Giné-Garriga et al., 2020), and it has a negative effect on QoL (Carrasco et al., 2022; Newman-Norlund et al., 2022). Similarly, pain is a risk factor for frailty (Lin et al., 2020; Saraiva et al., 2018) and a predictor of poor QoL and depression (Bonanni et al., 2023; Kaye et al., 2010).

Regarding the implications of the results, this study highlights which specific dimensions of QoL can be targeted for intervention, considering the distinct impacts of loneliness, frailty, and pain. For instance, recognizing that loneliness significantly affects control, autonomy, and pleasure suggests that interventions should focus on reducing feelings of loneliness. In this context, several studies have implemented strategies that alleviate loneliness and enhance QoL in older adults. For example, Richards et al. (2024) demonstrated that a technology-based program designed to develop digital skills in older individuals not only reduced social isolation and loneliness but also improved quality of life by enhancing self-management and self-efficacy. Similarly, Gould et al. (2021) found that a mobile intervention supported by therapists had the potential to improve mental health-related quality of life and reduce loneliness among older adults.

Regarding frailty, it was the main predictor to the dimension self-realization, and our study highlights the importance of prevention. Evidence suggests that physical activity and nutritional interventions can modify or even reverse frailty syndrome (Woolford et al., 2020). Moreover, multicomponent prevention programs, such as the one proposed by Yu et al. (2020), have demonstrated effectiveness in reducing frailty, as well as improving physical and cognitive function and self-rated health among community-dwelling older adults with pre-frailties. Given that frailty is a modifiable condition, these prevention programs should be considered for integration into community services for older adults.

## Figures and Tables

**Table 1:** Descriptive statistics and standardized factor loadings of the variables in the model.

Factor	Indicator	Range	$\bar{X}$	Md	SD	Factor Loading	S.D.	C.I.
Control	1	1–4	2.42	2	0.99	0.658	0.003	0.652, 0.664
	2	1–4	2.76	3	0.96	0.693	0.003	0.687, 0.699
	3	1–4	3.10	3	0.96	0.678	0.003	0.672, 0.684
Autonomy	1	1–4	3.15	3	0.88	0.501	0.004	0.494, 0.509
	2	1–4	3.14	3	0.95	0.169	0.005	0.160, 0.179
	3	1–4	2.61	3	1.14	0.390	0.004	0.381, 0.399
Pleasure	1	1–4	3.45	4	0.73	0.698	0.003	0.692, 0.704
	2	1–4	3.45	4	0.70	0.732	0.003	0.727, 0.738
	3	1–4	3.35	4	0.72	0.569	0.003	0.562, 0.575
Self-realization	1	1–4	2.99	3	0.86	0.747	0.002	0.743, 0.752
	2	1–4	2.97	3	0.88	0.780	0.002	0.776, 0.784
	3	1–4	2.92	3	0.87	0.797	0.002	0.793, 0.801
Frailty	Activity	1–4	3.29	4	1.06	0.359	0.009	0.341, 0.376
	Appetite	0–1	0.11	0	0.31	0.518	0.008	0.503, 0.534
	Fatigue	0–1	0.37	0	0.48	−0.455	0.005	−0.464, −0.446
	Strength	1–98	31.54	30	10.87	0.643	0.004	0.634, 0.651
	Slowness	0–1	0.22	0	0.41	−0.595	0.007	−0.608, −0.580
Pain	5 drugs	0–1	0.32	0	0.46	0.504	0.005	0.493, 0.514
	Drugs for pain	0–2	0.33	0	0.63	0.493	0.005	0.483, 0.503
Loneliness	1	1–3	1.42	1	0.66	0.606	0.007	0.593, 0.620
	2	1–3	1.33	1	0.58	0.780	0.005	0.770, 0.790
	3	1–3	1.25	1	0.52	0.798	0.005	0.788, 0.808

Note: S.D. = posterior standard deviation, C.I. = credibility interval, *p*-values are <0.001 for all factor loadings.

**Table 2:** Standardized results of the prediction effects and correlations of the variables included in the model.

Dependent Variables	Predictors	β	S.D.	*p*-Value	C.I.
Control	Frailty	−0.387	0.039	0.000	−0.464, −0.313
	Pain	−0.013	0.038	0.367	−0.085, 0.064
	Loneliness	−0.537	0.011	0.000	−0.559, −0.515
Autonomy	Frailty	−0.345	0.051	0.000	−0.448, −0.247
	Pain	−0.118	0.049	0.010	−0.211, −0.019
	Loneliness	−0.431	0.017	0.000	−0.465, −0.397
Pleasure	Frailty	−0.336	0.039	0.000	−0.415, −0.262
	Pain	0.089	0.038	0.007	0.018, 0.166
	Loneliness	−0.407	0.013	0.000	−0.432, −0.382
Self-realization	Frailty	−0.622	0.038	0.000	−0.701, −0.554
	Pain	0.076	0.037	0.012	0.010, 0.153
	Loneliness	−0.252	0.012	0.000	−0.274, −0.228
**Correlated Variables**		***r***	**S.D.**	***p*-Value**	**C.I.**
Control	Autonomy	0.752	0.015	0.000	0.721, 0.778
	Pleasure	0.099	0.014	0.000	0.072, 0.125
	Self-realization	0.318	0.013	0.000	0.293, 0.342
Autonomy	Pleasure	0.663	0.013	0.000	0.637, 0.688
	Self-realization	0.792	0.013	0.000	0.766, 0.817
Pleasure	Self-realization	0.717	0.006	0.000	0.705, 0.729
Frailty	Pain	0.875	0.009	0.000	0.859, 0.892
	Loneliness	0.506	0.009	0.000	0.487, 0.524
Pain	Loneliness	0.405	0.013	0.000	0.379, 0.429

Note: S.D. = posterior standard deviation, C.I. = credible interval.

## Data Availability

The data that support the findings of this study are available at the SHARE Research Data Center to the entire research community free of charge (www.share-project.org, accessed on 15 May 2022). Restrictions apply to the availability of these data, which were used under license for the current study and, thus, are not publicly available. Data are, however, available from the authors upon reasonable request and with permission of the SHARE Project (https://share-eric.eu/data/data-access, accessed on 17 June 2025).

## Supplementary Materials

The following supporting information can be downloaded at: https://www.mdpi.com/article/10.3390/ejihpe15070127/s1, Table S1: Correlations between the variables included in the model.

## Abbreviations

The following abbreviations are used in this manuscript:

QoL	Quality of life
