# Supplementary material for: A Bayesian Structural Equation Model to Predict Quality of Life in European Older Adults
Source: Eur J Investig Health Psychol Educ. 2025 Jul 9;15(7):127. doi: 10.3390/ejihpe15070127 (PMC12293711; doi:10.3390/ejihpe15070127)
Supplement: Supplementary file 1 [file ejihpe-15-00127-s001.zip › ejihpe-3601906-supplementary.pdf]

European Journal of
Investigation in Health, Psychology and Education

MDPI

Article

A Bayesian Structural Equation Model to Predict Quality of Life in European Older Adults

José M. Tomás*, Aitana Sanz and Zaira Torres

Department of Methodology for the Behavioral Sciences, University of Valencia, 46010 Valencia, Spain

* Correspondence: tomasjm@uv.es
* Author to whom correspondence should be addressed.

Supplementary Materials

Table S1. Correlations between the variables included in the model

	Con1	Con2	Con3	Aut1	Aut2	Aut3	Plea1	Plea2	Plea3	Self1	Self2	Self3	Frail1	Frail2	Frail3	Frail4	Frail5	Pain1	Pain2	Lon1	Lon2
Con2	.46**	1.00																			
Con3	.39**	.50**	1.00																		
Aut1	.28**	.26**	.26**	1.00																	
Aut2	.14**	.18**	.20**	.03**	1.00																
Aut3	.26**	.24**	.29**	.16**	.24**	1.00															
Plea1	.22**	.24**	.27**	.30**	.07**	.18**	1.00														
Plea2	.25**	.24**	.28**	.31**	.05**	.19**	.52**	1.00													
Plea3	.16**	.18**	.23**	.23**	.07**	.19**	.37**	.41**	1.00												
Self1	.43**	.37**	.35**	.39**	.03**	.19**	.40**	.41**	.33**	1.00											
Self2	.36**	.31**	.35**	.36**	.05**	.27**	.43**	.44**	.38**	.57**	1.00										
Self3	.38**	.34**	.36**	.37**	.06**	.29**	.45**	.46**	.40**	.57**	.65**	1.00									
Frail1	.27**	.26**	.24**	.32**	.00	.07**	.26**	.28**	.14**	.39**	.27**	.29**	1.00								
Frail2	-.16**	-.18**	-.16**	-.14**	-.02*	-.07**	-.18**	-.18**	-.10**	-.25**	-.17**	-.21**	-.18**	1.00							
Frail3	-.30**	-.26**	-.18**	-.21**	-.02	-.08**	-.18**	-.22**	-.13**	-.42**	-.23**	-.29**	-.24**	.25**	1.00						
Frail4	.22**	.19**	.16**	.17**	.02**	.08**	.14**	.14**	.08**	.26**	.21**	.21**	.26**	-.14**	-.22**	1.00					
Frail5	-.33**	-.28**	-.23**	-.26**	.01**	-.11**	-.19**	-.22**	-.12**	-.39**	-.29**	-.30**	-.47**	.19**	.30**	-.26**	1.00				
Pain1	-.23**	-.19**	-.15**	-.17**	.02**	-.06**	-.13**	-.14**	-.09**	-.27**	-.18**	-.20**	-.25**	.17**	.25**	-.15**	.30**	1.00			
Pain2	-.24**	-.18**	-.15**	-.16**	-.01*	-.15**	-.13**	-.13**	-.10**	-.26**	-.20**	-.21**	-.20**	.13**	.23**	-.20**	.28**	.25**	1.00		
Lon1	-.24**	-.25**	-.34**	-.18**	-.03**	-.14**	-.25**	-.27**	-.17**	-.25**	-.24**	-.27**	-.17**	.16**	.20**	-.21**	.19**	.13**	.13**	1.00	
Lon2	-.29**	-.34**	-.60**	-.24**	-.14**	-.28**	-.33**	-.31**	-.21**	-.32**	-.36**	-.35**	-.19**	.16**	.18**	-.15**	.23**	.11**	.17**	.44**	1.00
Lon3	-.27**	-.33**	-.50**	-.24**	-.08**	-.19**	-.31**	-.34**	-.21**	-.31**	-.30**	-.32**	-.24**	.18**	.22**	-.18**	.26**	.15**	.17**	.51**	.61**

Notes: Con= Control, Aut= Autonomy, Plea= Pleasure, Self =Self-Realization, Frail=Frailty, Lon= Loneliness. * indicates p< .05, ** p<.01, ***p<.001
